# Imaging in neuro-ophthalmology: An overview

**DOI:** 10.4103/0974-620X.53033

**Published:** 2009

**Authors:** Anupam Kumar Kakaria

**Affiliations:** Department of Radiology, Sultan Qaboos University Hospital, Muscat, Oman

**Keywords:** Neuro-ophthalmology, computed tomography, magnetic resonance imaging, angiography

## Abstract

Advances in neuroimaging and interventional techniques have revolutionized the early diagnosis, prognosis, and treatment of neuro-ophthalmic disorders. These techniques include computed tomography (CT), magnetic resonance imaging (MRI), CT and MR angiographic techniques, catheter digital subtraction angiography, functional MRI, positron emission tomography, and single photon emission computed tomography. In this review, the value of current techniques in the diagnosis, localization, and treatment of various neuro-ophthalmic disorders is described.

## Introduction

As a superficial organ, the eye is easily accessible to the ophthalmologist for assessment using clinical tests. The initial assessment of the globe, its optical properties, and the light conducting media is best done by direct ophthalmological examination. In case of opacity of the media, or other difficulties in evaluation of the globe directly, ultrasound provides excellent visualization of the orbital contents. However, the evaluation of neural pathways of vision is not possible using these resources. Although initial clinical assessment plays a vital role in assessment of the visual neural pathway, diagnostic imaging is required subsequently to assess the anatomy and pathology of the visual neural pathways. Before the advent of cross sectional imaging, radiological assessment was limited to relatively insensitive techniques such as conventional radiography or tomography. The investigations such as catheter angiography, cisternography, pneumoencephalography, and ventriculography offered limited visualization of intracranial structures. These investigations relied on secondary changes produced in the CSF spaces or vascular anatomy. In addition to being invasive, these investigations had poor sensitivity towards parenchymal pathology. With wider availability and utilization of computed tomography (CT) and magnetic resonance imaging (MRI), the imaging has an increasing role to play in neuro-ophthalmology. These two complementary techniques form the bedrock of imaging in neuro-ophthalmology. Catheter angiography maintains its position in modern imaging primarily limited to intervention and treatment rather than diagnosis. Functional MRI, positron emission tomography (PET), and single photon computed tomography (SPECT) are used mainly as problem solving tools and to provide a functional counterpart to the anatomical information provided by CT and MRI.[1]

## Indications

Common indications for which radiological imaging is requested in neuro-ophthalomology are as follows.[2]

- Visual loss, unilateral or bilateral
- Field defects and scotomas
- Anisocoria or ptosis
- Proptosis
- Diplopia or ophthalmoplegia
- Oscillopsia (for example, nystagmus)
- Ophthalmoscopic abnormalities (papilloedema, drusen)
- Pupillary defects

## Imaging modalities used

- Computed tomography (CT)
- Magnetic resonance imaging (MRI)
- CT and MR angiographic techniques
- Catheter DSA
- fMRI, PET, and SPECT

## Computed Tomography (CT)

**Figure 1:**
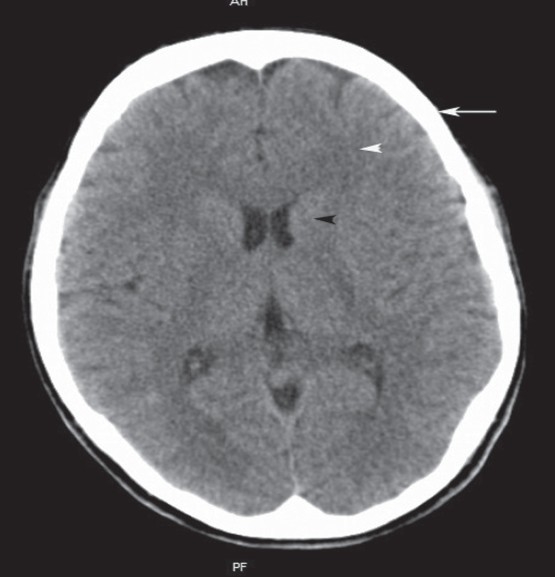
An image from CT of head showing gray matter (black arrowhead) denser than white matter (white arrowhead). Tissues with high calcium content like bone are very dense and appear white (arrow)

First results and descriptions of clinical CT were published in British journal of radiology in December 1973, by Hounsfield.[3] CT rapidly became an excellent modality for investigation of CNS, because of relatively static CNS structures and ability to immobilize the head. It depends on relative attenuation of the radiation beam by tissues with different density and atomic number. With rapid advances in computational technology and radiological hardware, it is now possible to image large volumes in very short time, effectively removing the deleterious effects of even physiological movements such as respiration and cardiac motion. Conventional CT was limited to scanning in one plane i.e., the axial plane and other planes had to be reconstructed using computer manipulation leading to loss of resolution in the Z axis. Multi slice helical CT allows acquisition of isometric volumes leading to spatial resolution in the sub mm range in all three planes, removing one of the big disadvantages of conventional CT [Figure 1].

## Magnetic Resonance Imaging (MRI)

**Figure 2:**
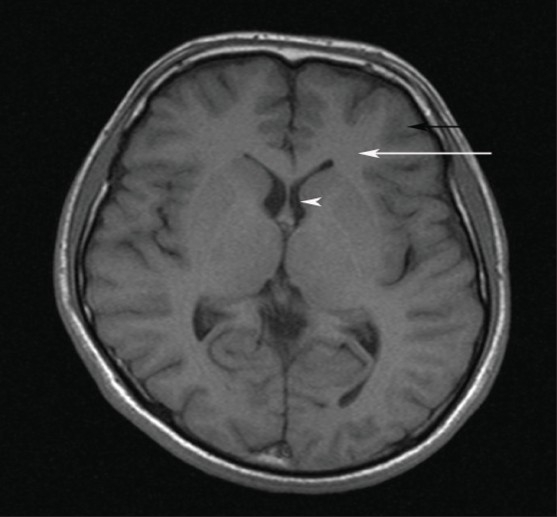
T1 weighted MR showing dark gray matter (black arrow), brighter white matter (white arrow) and dark CSF (white arrowhead)

**Figure 3:**
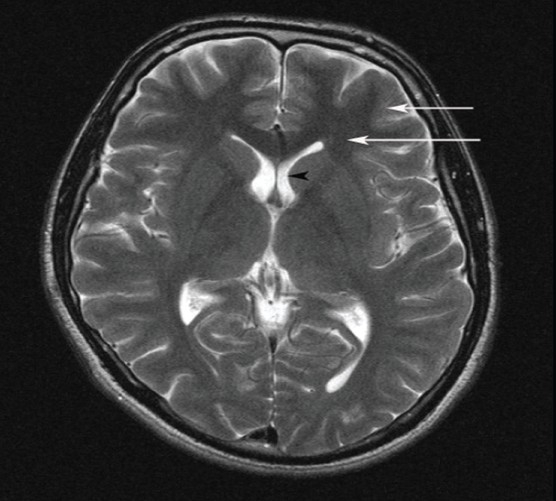
T2 weighted MR showing bright CSF (black arrowhead), dark white matter (long arrow) and brighter gray matter

**Figure 4:**
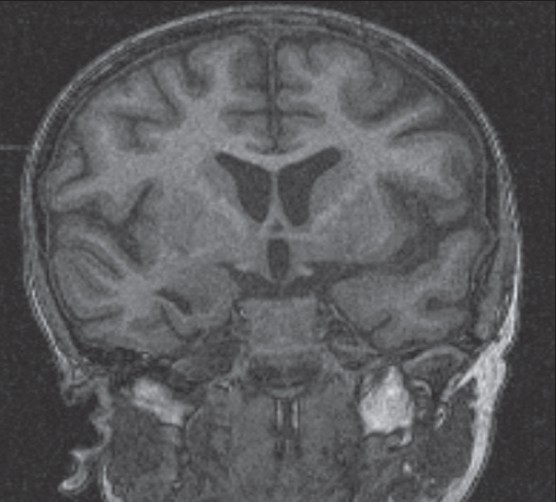
Thin section high resolution gradient echo MR image

**Figure 5:**
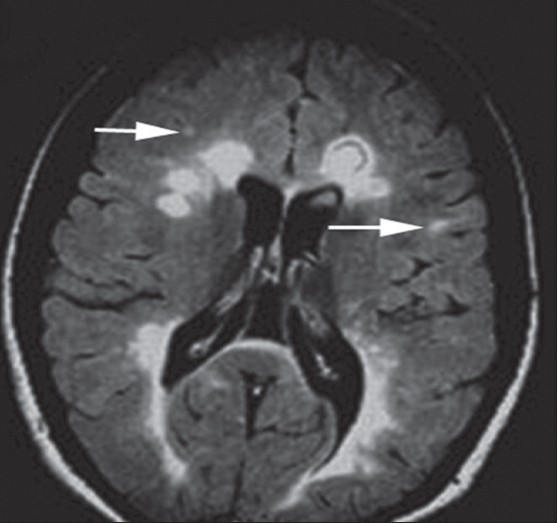
FLAIR MR image showing bright signal from the plaques of MS (white arrows)

The first principles of image formation by using nuclear magnetic resonance were published by Lauterbur in 1973.[4] Since those early days, there had been rapid development and great progress made in software and hardware capabilities of MRI to overcome many of its early limitations. Similar to CT, the initial work in MRI was predominantly in CNS. The reasons for this are also similar to CT that is the scanning time was very long extending in some cases up to 45 to 60 mins. However, rapid progress in computational capabilities has led to considerable reduction in imaging times. In current practice, MRI is the imaging modality of choice in all neuro-radiological imaging.[1,2,5] With dense bone surrounding the optic nerve at the apex of orbit, MRI provides unique possibilities of assessing the intrinsic lesions of the optic nerves and tracts. The study depends on interaction of protons (hydrogen nuclei) with a strong magnetic field in which they are placed. The CT was an extension of conventional imaging where it studied attenuation differences in tissues. The use of detectors and computers to enhance the difference in attenuation, changed the amount of information available, but did not change the kind of information available. The MRI has proven to be a paradigm shift in imaging as it involves fundamentally different principles and physics for assessment of anatomy and pathology. MRI offers the possibility of varying many factors thus opening up a large vista of different protocols and sequences and thus can have a long learning curve [Figures 2–5].

Although the advantages offered by MRI are many, there are certain areas where CT can be more useful such as, evaluation of bony trauma, evaluation of calcification, and in very sick patients who need rapid assessment [Table 1]. In spite of all attempts, there is a small number of patients who can not undergo MRI because of claustrophobia.[6]

**Table 1:** A brief comparison of computed tomography and magnetic resonance imaging capabilities

*Computed tomography*	*Magnetic resonance imaging*
Ionizing radiation	No ionizing radiation
Excellent visualization of acute hemorrhage	Difficult visualization of acute hemorrhage
Very sensitive to calcifi cation and bony lesions	Difficult visualization of calcifi cation and bony lesions
Limited planes	Multi planar imaging
Limited visualization near dense bone	Dense bone does not impose any limitation
Limited contrast resolution in soft tissues	Superb soft tissue contrast resolution
Quick to obtain, easily available, inexpensive	Limited availability, time consuming, expensive

Note: In addition to the above, the patients who are on life support systems are difficult to assess with MRI as most life support systems are not compatible with MRI. Special life support equipment compatible with MRI is available but is very expensive.

## CT and MR angiography

**Figure 6:**
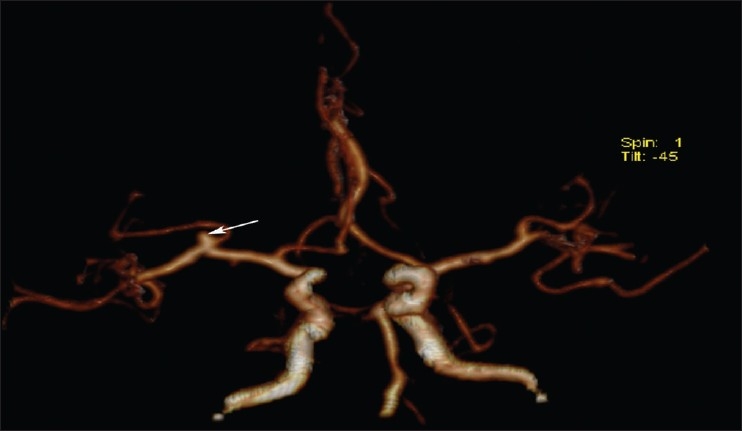
CT angiography showing a right middle cerebral artery aneurysm (white arrow)

**Figure 7:**
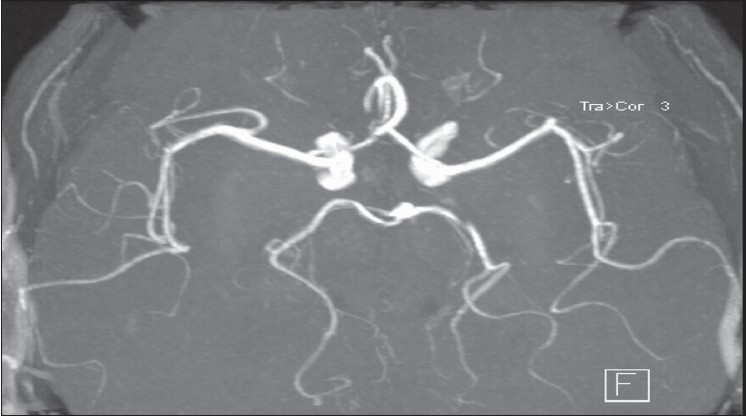
MR angiography showing normal vasculature at the skull base

The patients with suspected vascular abnormalities may undergo assessment using MR or CT angiographic techniques. The CT angiography entails assessment of arteries after intravenous injection of iodinated contrast; it provides anatomical information. On the contrary, MR depends on flowing blood to provide contrast, and MRA can be done with or without intravenous contrast [Figures 6 and 7].

## Diffusion weighted imaging and white matter tractography

**Figure 8:**
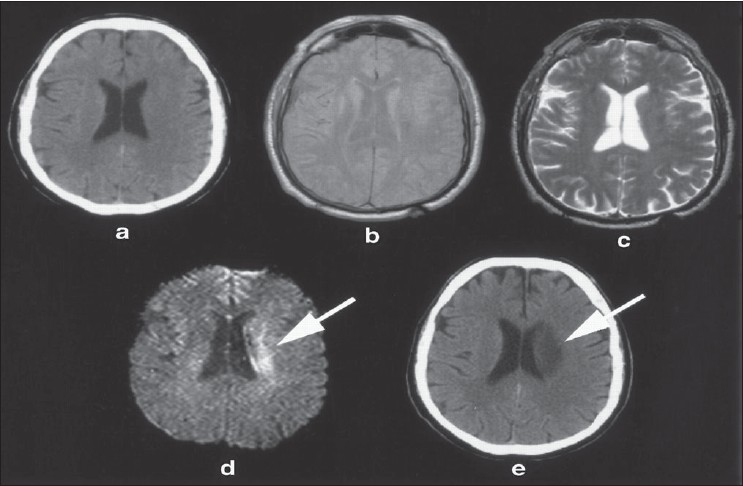
Composite image showing advantage of diffusion weighted MR in acute infarct. a, b, and c show normal CT, and conventional MR images not showing the infarct. Diffusion weighted image (d) shows an area of restricted diffusion in left anterior cerebral artery distribution consistent with an infarct. A CT at 48 hours, and (e) shows the infarct

The diffusion weighted uses the property of Brownian motion of free water molecules to assess the freedom with which the molecules can move in a particular tissue. Generally, the pathology is demonstrated by restriction of diffusion. An extension of this technique is used for white matter fiber tracking. In axons, the water is free to diffuse along the length of the white matter fiber tracks but diffusion is restricted at right angle to the length of the white matter tracts. This is called anisotropic diffusion. By acquiring diffusion weighted images in multiple planes it is possible to track the white matter fibers along their course. This technique has potential implications in assessment of the following: white matter tract connectivity in congenital anomalies; age related degenerative changes in white matter tracts; demyelinating conditions of white matter; neoplastic involvement and displacement of white matter tracts; and surgical treatment of CNS pathology while sparing the white matter tracts leading to reduced morbidity of the surgical procedure [Figure 8].[6-8]

## Catheter angiography and treatment

**Figure 9:**
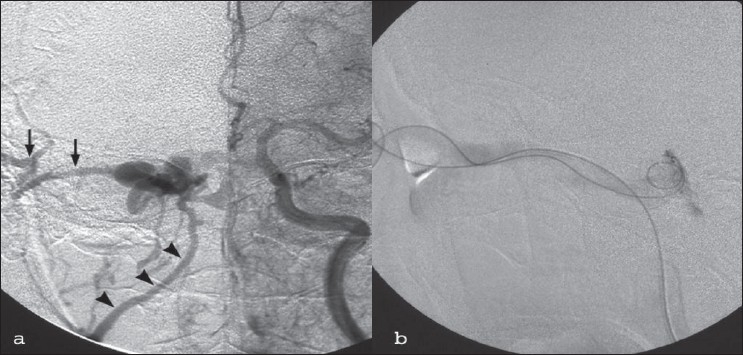
Catheter DSA in a patient with carotico cavernous fi stula. The black arrows and arrowheads in (a) show orbital veins, and in (b) show catheterization of fi stula prior to endovascular treatment

Prior to advent of the CT and MR angiography, catheter angiography formed the basis of all vascular assessment in the CNS. Besides being an invasive procedure, it has the potential to precipitate potentially devastating neurological sequelae. As a result, the CT and the MR angiographic techniques are supplanting catheter angiography for diagnostic purposes. However, with advances in catheter techniques and materials, catheter angiography has come to play a central role in endovascular management of many CNS vascular lesions with excellent results [Figure 9].[9]

### Guidelines

In general, MRI is the investigation of choice for most neuro-ophthalmological assessments. CT is better for evaluation of bony orbit in patients of trauma or suspected destructive osseous lesions. It is also superior for detection of presence of calcification such as in optic nerve drusen. The fact that MRI entails so many different variables leading to many sequences such as T1, T2 weighted, FLAIR, GRE, diffusion weighted imaging, spectroscopy, etc. means that the studies need to be tailored to the patients′ needs and clinical requirement. As in CT, IV contrast improves visualization of the lesions. Typically, the clinician need not worry about different sequences, as the imaging radiologist will decide which sequences will provide maximum information, depending on the patients′ clinical picture in the shortest time. This in turn places extra responsibility on the shoulder of the referring physician to provide complete and accurate information regarding the patients' clinical profile, as it will determine what imaging the patient should undergo. Typically, the clinical information must include the site of suspected lesion and if possible the nature of suspected lesion. The information on site is important to allow detailed study of the relevant area. As detailed study of multiple areas in one examination ends up adding the time required for each examination individually; which leads to a very long and time consuming examination which in turn reduces the patient cooperation. Another potential down side in trying to cover a large area is reduced resolution in an attempt to reduce time of examination. The information regarding the nature of the suspected pathology is important in deciding the protocol of examination and if intravenous contrast is required. In case of any doubt or confusion regarding which examination would best serve the interests of the patient, the best course of action would be to contact the radiologist. Direct interaction with the radiologist will reduce the potential for miscommunication and allow the radiologist to ask for supplementary information and give his suggestions regarding the best course of action.

### Common errors

Wolintz *et al*., in 2004 reviewed their case material for assessing the errors in use of MRI for neuro-ophthalmic disorders.[10] According to them the errors could be divided into prescriptive and interpretive errors. Each of these categories could further be divided into four types.

The four prescriptive errors were:

- Failure to apply a dedicated study.
- Inappropriate application of a dedicated study.
- Omission of intravenous contrast.
- Omission of specialized sequences.

The interpretive errors were:

- Failure to detect a lesion because of misleading clinical information.
- Rejection of a clinical diagnosis because an expected imaging abnormality was absent.
- Assumption that a striking abnormality accounted for the clinical abnormality.
- Failure to consider lack of clinical specificity of the imaging abnormalities.

After reviewing the above points it is clear that most of the errors resulted directly from inadequate communication between the referring clinician and the performing and interpreting radiologist.

### Suggestions for improving the results

The most common point of dispute is when either the imaging is normal in face of definite clinical signs or the imaging finding is not able to explain the clinical profile of the patient. In these cases the solution is to call the radiologist and discuss the case. Preferably, the neuro-ophthalmologist and the radiologist should together see the images and ensure the adequacy of the study. In such cases, the clinician brings his clinical acumen and possibly additional clinical data to the table and then the radiologist can make sure that the clinically relevant areas are imaged adequately without any artifacts. In case, the clinical symptomatology is progressive, it is also appropriate during this discussion to see if additional imaging with different imaging parameters such as different magnification or slice thickness will improve or add to the quality of imaging. Finally the referring clinician must realize that the images are representation of different processes in the patient and could be normal in face of definite clinical findings.[11]

## Teaching points

An imaging study is only as good as the clinical information provided to the radiologist.

Communication between the referring clinician and the radiologist is the single most important problem solving mechanism in difficult or complicated cases.
